# Evaluating Self-declared Ancestry of U.S. Americans with Autosomal, Y-chromosomal and Mitochondrial DNA

**DOI:** 10.1002/humu.21366

**Published:** 2010-12

**Authors:** Oscar Lao, Peter M Vallone, Michael D Coble, Toni M Diegoli, Mannis van Oven, Kristiaan J van der Gaag, Jeroen Pijpe, Peter de Knijff, Manfred Kayser

**Affiliations:** 1 Department of Forensic Molecular Biology, Erasmus MC University Medical Center RotterdamRotterdam, The Netherlands; 2 National Institute of Standards and Technology, Biochemical Science Division100 Bureau Drive, Mail Stop 8311, Gaithersburg, MD 20899-8311, United States of America, USA; 3 Research Section, The Armed Forces DNA Identification LaboratoryRockville, Maryland, United States of America, USA; 4 Department of Human and Clinical Genetics, Leiden University Medical CenterLeiden, The Netherlands

**Keywords:** U.S. Americans, genetic ancestry, self-declared ancestry, ASM, AIM, Y-chromosome, NRY, mtDNA

## Abstract

The current U.S. population represents an amalgam of individuals originating mainly from four continental regions (Africa, Europe, Asia and America). To study the genetic ancestry and compare with self-declared ancestry we have analyzed paternally, maternally and bi-parentally inherited DNA markers sensitive for indicating continental genetic ancestry in all four major U.S. American groups. We found that self-declared U.S. Hispanics and U.S. African Americans tend to show variable degrees of continental genetic admixture among the three genetic systems, with evidence for a marked sex-biased admixture history. Moreover, for these two groups we observed significant regional variation across the country in genetic admixture. In contrast, self-declared U.S. European and U.S. Asian Americans were genetically more homogeneous at the continental ancestry level. Two autosomal ancestry-sensitive markers located in skin pigmentation candidate genes showed significant differences in self-declared U.S. African Americans or U.S. European Americans, relative to their assumed parental populations from Africa or Europe. This provides genetic support for the importance of skin color in the complex process of ancestry identification. © 2010 Wiley-Liss, Inc.

## INTRODUCTION

The current U.S. American population is particularly interesting for studying bio-geographic ancestry, as it represents an amalgam of individuals who originate from at least four major continental regions that (at least potentially) started to admix at different time scales from the first European colonization of North America onwards. The four most frequently self-assigned clusters by U.S. Americans according to the U.S. Census Bureau (2008) are White (U.S. European), Black (U.S. African), Asian (U.S. Asian) and Hispanic / Latinos (U.S. Hispanic). It should be noticed, however, that such classification mixes bio-geographic ancestry with sociological and cultural, including linguistic variables. For example, individuals self-defined as U.S. Hispanics share cultural aspects, such as the Spanish mother tongue, but can be of different bio-geographic ancestry reflecting the more than 500 years of admixture history between Native Americans, Europeans and Africans in the Americas (Salazano and Bortolini, 2002). Similarly, self-declared U.S. Africans generally carry some degree of European genetic ancestry which in particular cases can reach more than 80% of the total ancestry (Sinha, et al., 2006). Finally, additional sub-continental population substructure can also be detected within self-identified groups, such as within U.S. Europeans (Campbell, et al., 2005), U.S. Africans (Tishkoff, et al., 2009; Zakharia, et al., 2009) and U.S. Hispanics (Wang, et al., 2008), as genetic heterogeneity within the respective parental populations has also been observed (Jakobsson, et al., 2008; Lao, et al., 2008; Li, et al., 2008; Novembre, et al., 2008).

In the present study we have analyzed the bio-geographic ancestry of U.S. Americans with self-declared African, European, Asian and Hispanic ancestry, respectively, using single nucleotide polymorphisms (SNPs) from uniparental non-recombining part of the human Y-chromosome (NRY) and mitochondrial (mt) DNA, as well as from carefully ascertained biparental autosomal regions. All DNA markers used were ascertained to be sensitive for indicating bio-geographic ancestry on the level of the four continental regions (Africa, Europe, Asia, and America) expected to have contributed to the current U.S. population. Very few previous studies have analyzed all three genetic systems in at least one of these U.S. groups (Parra, et al., 1998; Lind, et al., 2007; Stefflova, et al., 2009). As far as we know, our study represents the first of its kind combining suitable ancestry-sensitive markers from all three genetic systems to detect separately patrilineal, matrilineal and biparental genetic ancestry in all four major U.S. American groups.

## MATERIALS AND METHODS

### Samples

Anonymous liquid blood or buccal swab samples from a total of 664 U.S. American individuals were obtained from Interstate Blood Bank, Inc. (Memphis, TN), Millennium Biotech, Inc. (Ft. Lauderdale, FL) and DNA Diagnostics Center (Fairfield, OH). Among them, 246 were self-declared U.S. African Americans, 127 were self-declared U.S. Hispanic Americans, and 245 were self-declared U.S. European Americans from Temple and Killeen, TX, Louisville, KY, Baltimore, MD, Philadelphia, PA, Memphis, TN and Miami, FL and 46 were self-declared U.S. Asian Americans from the Fairfield, OH source. Each sample was examined with 15 autosomal short tandem repeats and the amelogenin sex-typing marker using the AmpFlSTR Identifiler kit (Applied Biosystems, Foster City, CA) to verify that each sample was unique (Butler, et al., 2003; Decker, et al., 2008). In addition to the U.S. American samples, autosomal markers were also genotyped in the Human Genome Diversity Project- Centre d'Etude du Polymorphisme Humain (HGDP-CEPH) samples (Cann, et al., 2002). From those, four groups i.e. i) Sub-Saharan Africans (Bantu, Biaka Pygmies, Mandenka, Mbuti Pygmies, San, Yoruba); ii) East Asians (Cambodian, Dai, Daur, Han, Hazara, Hezhen, Japanese, Lahu, Miaozu, Mongola, Naxi, Oroqen, She, Tu, Tujia, Uygur, Xibo, Yakut, Yizu); iii) Eurasians (Adygei, Basque, Bergamo, French, Orcadian, Russian, Sardinian, Tuscan); and iv) Native Americans (Colombian, Karitiana, Maya, Pima, Surui) were used as parental groups in some of the statistical analyses.

### Autosomal DNA analysis

Tweny four autosomal SNPs: rs1876482, rs2179967, rs1048610, rs1371048, rs1478785, rs1369290, rs952718, rs1405467, rs1344870, rs1391681, rs1461227, rs1907702, rs2052760, rs714857, rs721352, rs722869, rs926774, rs1448484, rs1667751, rs1858465, rs1465648, rs16891982, rs1808089, rs3843776 were genotyped via two SNaPshot multiplex reactions as described in detail in the Supp. Methods and Supp. Table S1. These SNPs were ascertained to be ancestry-sensitive on the continental level as described in detail elsewhere (Lao, et al., 2006; Lao, et al., 2007; Kersbergen, et al., 2009; Corach, et al., 2010). In brief, Affymetrix 10K SNP data in 76 human individuals from 21 worldwide sampling localities from the Y-Chromosome Consortium (YCC) panel were analyzed using the informativeness of ancestry statistic (*I_n_*; (Rosenberg, et al., 2003)) and applying a genetic algorithm to select a minimal set of markers that maximized the amount of ancestry information for differentiating four continental populations (Sub-Saharan Africa, Eurasia, East Asia and America) (Lao, et al., 2006). In parallel, a single population F_ST_ (Weir and Cockerham, 1984) strategy was applied to ascertain markers that differentiate each population (Kersbergen, et al., 2009). In addition, SNPs were added from 3 genes associated with variation in skin pigmentation showing large frequency differences between Europeans, Africans and East Asian ancestry and for which evidence of positive selection was established (Lao, et al., 2007). The current set of 24 ancestry-sensitive markers (ASMs) was obtained by ascertaining from the pooled data the set of SNPs that maximizes the *I_n_* statistic considering four continental groups.

### Mitochondrial DNA analysis

The entire mtDNA control region [range 16024-576] was sequenced using an automated, high-throughput, redundant sequencing and review strategy as described elsewhere (Irwin, et al., 2007). Sequence assembly and confirmation was performed independently by two different analysts, and followed by electronic data transfer to a secured laboratory information management system (LIMS) for sequence verification. The raw data was then exported to a second laboratory (the European DNA Profiling Group (EDNAP) mtDNA Population Database (EMPOP); (Parson and Dur, 2007)) for additional review and quality control examination. Control region haplotypes for the self-declared African American (Diegoli, et al., 2009) and Hispanic (Saunier, et al., 2008) samples have been published previously, and the sequences, along with those generated here for European Americans and Asian Americans have all been deposited in GenBank under accession numbers: DQ906460-DQ906701 and DQ906703-DQ906708 (African Americans), DQ906175-DQ906459 (European Americans), EU014897-EU015024 (Hispanics), and HM214959-HM215005 (Asian Americans). MtDNA haplogroup assignment of the samples was conducted using a multitude of references found within the reference section of (Diegoli, et al., 2009) for the African American samples, (Saunier, et al., 2008) for the Hispanic samples, (Irwin, et al., 2008) for the European American samples, and (Irwin, et al., 2009) for the Asian American samples, and checked against the most recent human mtDNA tree at http://www.phylotree.org (van Oven and Kayser, 2009). In those cases where haplogroup assignment based upon sequence polymorphisms in the control region was ambiguous, additional sequencing of coding region SNPs was performed as described elsewhere (Just, et al., 2008). The continental region of geographic origin of the mtDNA haplogroups was assumed from published mtDNA data (Richards, et al., 1998; Macaulay, et al., 1999; Finnila, et al., 2001; Kivisild, et al., 2006; Kong, et al., 2006; Achilli, et al., 2008; Behar, et al., 2008), and is provided for all mtDNA haplogroups observed in this study in Supp. Table S2.

### Y-chromosomal DNA analysis

**Supp. Figure S1:**
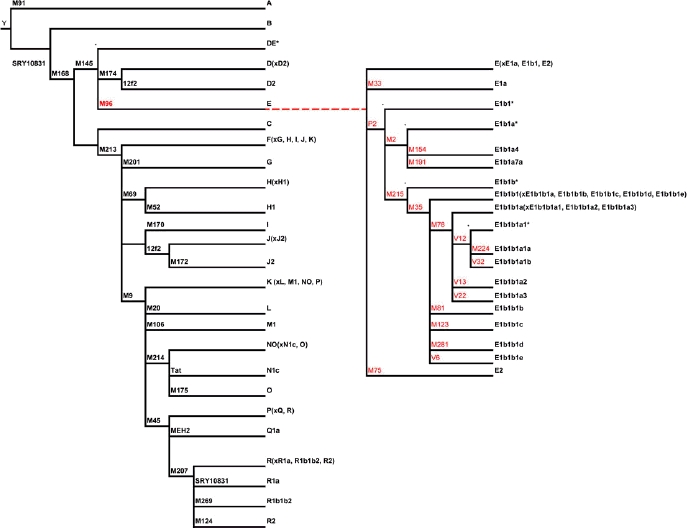
Phylogenetic tree of NRY SNPs.

Y-chromosome variation was identified by means of 42 NRY-SNPs in total. Twenty four NRY-SNPs were genotyped in all samples (including: SRY 1532, M91, M168, M145, M174, 12f2, M96, M213, M201, M69, M52, M170, M172, M9, M20, M106, M214, Tat, M175, M45, MEH2, M207, M269, and M124). Aiming to maximize continental differentiation of haplogroup origins we additionally genotyped 18 additional SNPs among samples identified as belonging to haplogroup E (M33, P2, M2, M154, M191, M215, M35, M78, V12, M224, V32, V13, V22, M81, M123, M281, V6, and M75). A single multiplex PCR and SNaPshot assay using the principle of primer extension was designed for the core set of 24 NRY-SNPs as described elsewhere (Corach, et al., 2010). Genotyping of the additional 18 NRY SNPs for subtyping of haplogroup E was performed in a multiplex, designed in a similar way as described for the core set of 24 NRY-SNPs, the only exception being a final MgCl_2_-concentration of 3mM in the multiplex PCR. PCR-product sizes ranged from 76-150 bp. Sequences and concentrations of the primers used in the monoplex and multiplex PCR and extension reactions are provided in Supp. Table S3 and a phylogenetic tree of the NRY-SNPs used is in the Supp. Figure S1. NRY haplogroups were derived from genotyping of NRY-SNPs using the marker phylogeny as described elsewhere (Karafet, et al., 2008). The continental region of geographic origin of the NRY haplogroups was assumed from published NRY data (Semino, et al., 2000; Bortolini, et al., 2003; Jobling and Tyler-Smith, 2003; Luis, et al., 2004; Cruciani, et al., 2007), and is provided for all NRY haplogroups observed in this study in Supp. Table S4.

### Statistical analyses

Suitability of the 24 ascertained SNPs to recover continental ancestry was checked by means of performing a STRUCTURE analysis (Pritchard, et al., 2000) in the HGDP-CEPH panel. We increased the number of groups from K=2 to K=6 under the Admixture model with a burn-in of 100,000 simulations and retaining the next 100,000. Five runs were performed for each K. For the estimation of the parental ancestry of the U.S. samples, a STRUCTURE analysis considering four parental populations (Native Americans, East Asians, Eurasians, and Sub-Saharan Africans from HGDP-CEPH) based on expected continental ancestry was used. Ten thousand simulations were used as burn-in and the next 10,000 simulations retained for admixture estimates. Reproducibility of results was checked by repeating 10 times the same analyses, obtaining in all cases similar values of admixture from the parental populations. Bar plot was performed from the STRUCTURE estimations with Distruct software 1.1 (Rosenberg, 2004). Differences in the amount of ancestry were tested in regions with more than 10 sampled individuals by means of a Kruskal-Wallis test. In particular, it was computed for the African component in U.S. Africans (regions = Baltimore (n = 34), Louisville (n = 21), Memphis (n = 41), Miami (n = 25), Philadelphia (n = 104), Temple (n = 17)) and for the Native American component in U.S. Hispanics (regions = Miami (n=61), Temple (n=29), Killeen (n=17), Philadelphia (n=13)). Additionally, we compared the genetic clustering of U.S. individuals with self-identified ethnicity by means of a STRUCTURE analysis assuming no admixture between the inferred clusters and 4 populations (Tang, et al., 2005). An identical by state distance matrix between all pairs of individuals including parental HGDP-CEPH populations was computed considering the 24 SNPs and was used to compute a non parametric multidimensional scaling (MDS) (Kruskal and Wish, 1990) with the package isoMDS of the R software (R Development Core Team, 2006) specifying 3 dimensions. When the distance between two individuals was 0, a small quantity of 0.001 was added. The *I_n_* statistic was computed for each of the 24 ASMs using as populations: self-declared U.S. European and the Sub-Saharan African HGDP-CEPH population cluster (set A), self-declared U.S. African and HGDP-CEPH European group (set B), and Sub-Saharan African HGDP-CEPH population cluster and HGDP-CEPH European group (set C). A linear regression was performed with SPSS (SPSS, 2003) between set A and C, and between set B and C; the SNPs falling out of the prediction with a 99% confidence estimation in any of the two linear regressions were recovered. Analysis of Molecular Variance (AMOVA; (Excoffier, et al., 1992)) was conducted in Arlequin 3.0 software (Excoffier, et al., 2005) assuming self-identified ancestry.

## RESULTS

### Autosomal DNA

**Figure 1:**
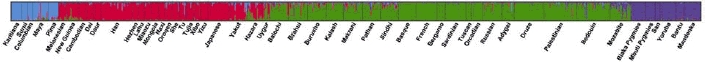
Genetic ancestry per individual in the global HGDP-CEPH panel as estimated by STRUCTURE using 24 autosomal ASMs (K=4).

The ancestry information provided by the 24 autosomal ASMs was first tested by performing a STRUCTURE analysis with the HGDP-CEPH samples assuming no prior knowledge of the ancestral groups. After K=4 the estimated loglikelihood of the data given the model (-19135) did not substantially change anymore. The four clusters detected at K=4 broadly match the four geographic regions: America, Sub-Saharan Africa, East Asia, and Eurasia (including Europe / Middle East / South Asia / Central Asia) (Figure 1). Only a small percentage of misclassified individuals was observed i.e., 0.47% Sub-Saharan Africans, 4.2% of Eurasians, 4.6% of Native American individuals, and 6.2% of East Asians (the latter was mainly in the Eurasian cluster with 3.6%). We concluded that these 24 SNPs are suitable for inferring bio-geographic ancestry in U.S. Americans since the four geographic regions identified represent the putative parental populations of the four major groups of U.S. Americans.

**Figure 2:**
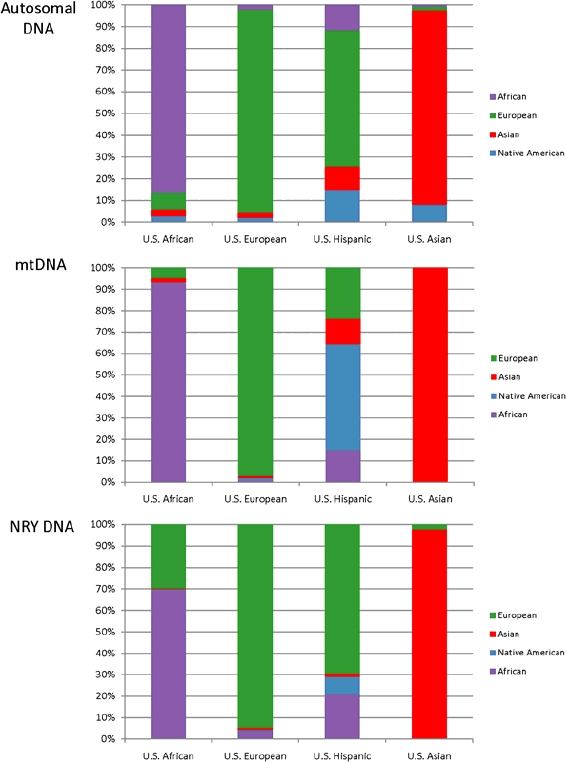
Proportions of average continental genetic ancestry in four U.S. American groups of self-declared ancestry based on autosomal DNA, mtDNA and NRY DNA.

Next, we used the Native Americans, East Asians, Eurasians, and Sub-Sahara Africans from HGDP-CEPH as parental groups of the U.S. Americans (the genotype data of the 24 autosomal SNPs can be found in the Supp. Table S5) in a STRUCTURE analysis. Self-declared U.S. Europeans showed on average 93.2% of European ancestry (95% CI from 73.23% to 98.09%), self-declared U.S. Asians carried on average 89.5% of East Asian ancestry (95% CI from 37.43% to 97.46%), and self-declared U.S. Africans revealed on average 86.2 % Sub-Sahara African ancestry (95% CI from 47.82% to 98.5%) (Figure 2). For these three U.S. groups rather small (between 0.8 and 8.1% on average) components of continental ancestries other than the self-declared ones were detected (Figure 2). In contrast, self-declared U.S. Hispanics carried on average 61.2% European ancestry (95% CI from 8.33% to 95.75%), 14.9% Native American (95% CI from 1.21% to 55.54%), 10.8% East Asian (95% CI from 1.12% to 56.35%), and 11.6%, Sub-Saharan African ancestries (95% CI from 0.41% to 58.49%) (Figure 2). Furthermore, we observed for self-declared U.S. Africans statistically significant heterogeneity in the amount of African genetic ancestry depending on the geographic sampling region (Kruskal-Wallis test p-value=0.0042), as well as for self-declared U.S. Hispanics in the amount of Native American genetic ancestry (Kruskal-Wallis p-value = 1.48e-07). An AMOVA grouping individuals based on self-declared ancestry explained 34.2% (two tail p value <0.0005) of the total genetic variation suggesting strong genetic differentiation between self-declared ancestry groups of U.S. Americans.

Furthermore, we performed an additional STRUCTURE analysis considering only U.S. samples with K=4 and assuming no admixture (loglikelihood of the data given the model = -16287.9) showing that the majority of U.S. Africans appeared in one of the four clusters (K4), and almost all U.S. Asians were in another cluster (K1) (see Table 1). In contrast, 15% of self-declared U.S. Hispanic samples were classified in the main cluster of U.S. Europeans (K3), and 19% of self-declared U.S. Europeans were clustered in the main cluster of self-declared U.S. Hispanics (K2).

**Table 1:** Correspondence between self-declared ancestry and STRUCTURE-based genetic ancestry inferred from 24 autosomal ASMs in four major U.S. American self-declared groups

	Clusters from STRUCTURE			
Self-declared ancestry	K1	K2	K3	K4
U.S. African	0%	2.2%	1.0%	96.8%
U.S. European	0%	19.0%	80.6%	0.4%
U.S. Hispanic	2.4%	77.8%	15.7%	4.0%
U.S. Asian	99.9%	0.1%	0%	0%

**Figure 3:**
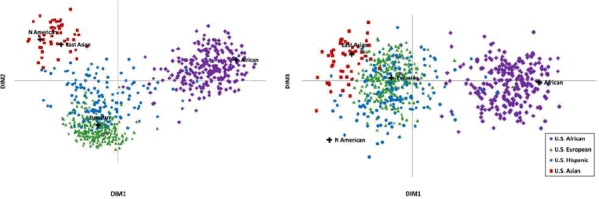
Two-dimensional plots of the first dimension, second dimension and third dimension obtained from a MDS analysis (stress = 0.13) performed with an Identical By State (IBS) distance matrix computed between pairs of individuals. Centroids of the four continental parental populations from HGDP-CEPH are marked by crosses.

From the MDS plot (Figure 3) it is evident that self-declared U.S. Europeans, U.S. Africans and U.S. Asians form rather discrete data clouds without strong overlaps between these groups, and tend to cluster close to their respective continental parental populations (from HGDP-CEPH). Self-declared U.S. Hispanics, however, did not cluster separately but either overlapped with U.S. / continental Europeans or appear between the U.S. / continental European cluster and the U.S. / continental Asian cluster with some U.S. Hispanics overlapping with the U.S. / continental African cluster or appeared between the U.S. / continental African and the U.S. / continental European clusters.

We also tested whether any of the 24 autosomal ASMs were more or less informative proportionally to the amount of information of the other markers for self-identification of U.S. Africans and U.S. Europeans. The lineal regression between the *I_n_* values computed for each SNP using U.S. Europeans and continental Africans (from HGDP-CEPH) versus continental Africans and continental Europeans (from HGDP-CEPH) (see methods for definition of continental populations) was highly statistically significant (R-squared = 0.98, two tail p-value = 3.91e-020; slope = 1.07, p value different from one = 0.0375). The *I_n_* value observed for rs16891982 when considering U.S. Europeans and continental Africans was significantly higher (falling out of the 99% predicted interval) than the one predicted by the linear regression using all 24 markers. In a similar way, comparison of the *I_n_* values computed between U.S. Africans and continental Europeans versus these computed considering continental Africans and continental Europeans also was statistically significant (R-squared = 0.97, two tail p-value = 1.85e-018; slope = 0.67, p value that the slope is different from 1 = 3.04e-12). Rs1448484 showed a larger *I_n_* value and rs16891982 smaller for the comparison between U.S. Africans and continental Europeans than predicted by the linear regression considering all 24 markers.

### NRY-DNAandmtDNA

The values of genetic ancestry provided by uni-parentally inherited NRY and mtDNA markers (Figure 2) were similar to the autosomal ASMs in the case of self-declared U.S. Europeans (estimated European ancestry for NRY: 94.7% and mtDNA: 96.7%; Fisher exact test value of the hypothesis of equal proportion of ancestry components between NRY and mtDNA = 4.85, two tail p value = 0.19) and for U.S. Asians (estimated East Asian ancestry for NRY: 97.8% for NRY and mtDNA; Fisher exact test value = 1.40, two tail p value = 1). In contrast, self-declared U.S. Africans showed discrepancies between the three genetic systems: 69.5% of NRY-DNA but 92.7% of mtDNA were of African ancestry and the second largest NRY ancestry component was European with 29.7%. The differences in the ancestry proportions between the two types of uniparental markers in U.S. Africans were highly statistically significant (Fisher exact test value = 58.80, two tail p value = 6.00e-014). In contrast to autosomal ASMs, we did not detect any statistically significant geographic substructure in the NRY and mtDNA ancestry data within self-declared U.S. Africans (Fisher statistic for NRY = 22.82, two tail p-value = 0.45 and Fisher statistic for mtDNA = 19.56, two tail p-value = 0.39). Self-declared U.S. Hispanics, however, showed the most complex ancestry pattern of all the U.S. American groups studied also for uniparental markers. NRY ancestry was 69.3% European, 21.3% African and only 7.9% Native American, whereas the East Asian component was 1.6%. MtDNA ancestry was 48.8% Native American, 23.6% European and 11.8% East Asian. Differences on ancestry proportions in U.S. Hispanics between the two uni-parentally inherited marker systems were statistically significant (Fisher exact test value = 82.41, two tail p value = 3.11e-018). In contrast to autosomal ASMs, there was no significant NRY differentiation between self-declared U.S. Hispanics from the different sampling regions across the country (Fisher statistic for NRY = 11.69, two tail p-value = 0.14), whereas mtDNA data revealed statistically significant differences (Fisher statistic for mtDNA = 23.3, two tail p-value = 0.0024) as autosomal ASMs did. AMOVA analyses performed on the NRY and mtDNA data separately and considering self-declared ancestry grouping explained 27.65% (two tail p value < 0.000005) and 7.6% (two tail p value < 0.000005) of the total genetic diversity, respectively. AMOVA using the autosomal ASM data and considering groupings based on NRY ancestry and separately on mtDNA ancestry revealed 23.3% (two tail p-value <0.0005) and 30.2% (two tail p-value <0.0005) of the total genetic diversity, respectively. The NRY and mtDNA haplogroups for all individual samples included can be found in the Supp. Table S5.

## DISCUSSION

The current U.S. population represents a mixture of groups with different bio-geographic ancestries, mainly from Europe, Sub-Saharan Africa, East Asia and the Americas. We have shown in the HGPD-CEPH samples that the ascertained autosomal ASMs are informative for detecting the ancestry of these four continental groups. Overall, STRUCTURE, MDS and AMOVA analyses indicate that in U.S. Americans self-declared ancestry serves on average as a good proxy of the underlying autosomal genetic diversity, especially of European, African and Asian Americans. Our STRUCTURE results are in line with an earlier study reporting that ancestry self-identification corresponded well with STRUCTURE-based predictions for U.S. Americans (Tang, et al., 2005). Our findings with autosomal ASMs tend to corroborate previous findings performed in self-identified U.S. Europeans (Halder, et al., 2008; Halder, et al., 2009; Kosoy, et al., 2009) and U.S. Asians (Kosoy, et al., 2009), although usually many more markers were applied before. However, we observed discrepancies between our data and previous studies for self-declared U.S. Africans and U.S. Hispanics. For U.S. Africans we found a slightly larger percentage of African ancestry and a slightly lower percentage of European ancestry relative to previous reports (Tian, et al., 2006; Halder, et al., 2008; Halder, et al., 2009; Kosoy, et al., 2009; Zakharia, et al., 2009). For U.S. Hispanics, the Native American component tends to be rather low compared to previous studies (Price, et al., 2007; Halder, et al., 2009; Kosoy, et al., 2009). Differences in the admixture histories in different regions of the U.S. as reported elsewhere (Salazano and Bortolini, 2002; Kittles and Weiss, 2003; Zakharia, et al., 2009) are likely to explain such discrepancies. This view also is supported by the considerable heterogeneity in continental genetic ancestry depending on the geographic origin of the sampling region within the U.S. we observed for these two U.S. American groups. An alternative explanation in the case of U.S. Hispanics could be a lack of power of the set of autosomal ASMs we applied to distinguish Native American from East Asian ancestry (also explaining the apparent small Native American ancestry component in U.S. Asians). Native Americans and East Asians show a general genetic proximity due to their shared population history (Jakobsson, et al., 2008; Li, et al., 2008). Repeating the STRUCTURE analysis for U.S. Hispanics without considering East Asians as parental population raised the Native American ancestry component up to 27.44%, which is more comparable to previous studies. However, the fact that some of the self-declared U.S. Hispanic individuals carried NRY haplogroups typical for East Asians, and because a previous study also detected Asian ancestry in U.S. Hispanics (Guthery, et al., 2007), indicate that excluding East Asian admixture a priory would be incorrect for estimating genetic ancestry in U.S. Hispanics.

Ancestry estimations obtained here with uni-parentally inherited markers are in good agreement with previous studies for U.S. Europeans, U.S. Africans and U.S. Hispanics for NRY (Kayser, et al., 2003; Hammer, et al., 2006; Lind, et al., 2007) and mtDNA (Allard, et al., 2002; Allard, et al., 2004; Allard, et al., 2005). In contrast, the percentage of Native American mtDNA ancestry estimated in the U.S. Hispanics studied here appears smaller than that of other studies (ranging from ∼70% to ∼85.11%) (Merriwether, et al., 1997; Allard, et al., 2006), although differences between U.S. Hispanic groups from different U.S. regions were observed, which may explain the discrepancies

Combining the ancestry information of patrilineal, matrilineal and biparental markers, a special quality of our study, offers the possibility to study the patterns of admixture at different levels of complexity. We observed the same degree of ancestry homogeneity in the three types of genetic markers for self-identified U.S. Europeans and U.S. Asians, which suggests relatively low genetic admixture with other ancestry groups than the one indicated by self-declaration. Noticeably, this finding for U.S. Europeans contrasts with common observation for self-declared European Americans from South America (Goncalves, et al., 2007; Corach, et al., 2010). In those South American groups European ancestry signals are usually high for NRY-DNA, intermediate for autosomal DNA, but low for mtDNA, whereas Native American genetic ancestry signals are reverse, indicating sex-bias admixture between mostly European men and mostly Native American women (Goncalves, et al., 2007; Corach, et al., 2010). This discrepancy between European Americans from North and South Americans has been explained in terms of local differences in social practices (Goncalves, et al., 2007). However, it could also be explained if the concept of ancestry self-identification had different meanings depending on the region of residence. This is supported by the fact that genetic admixture proportions of self-identified U.S. Hispanics from our study resemble those from self-declared European Americans in some South American countries with similar evidence for sex-biased admixture history. Our data also indicate sex-biased admixture for U.S. Africans with considerably more European NRY than mtDNA ancestry, and autosomal DNA estimates in-between. Previous studies analyzing NRY and mtDNA ancestry in U.S. Africans have reported similar results (Kayser, et al., 2003; Lind, et al., 2007), (see (Stefflova, et al., 2009) for a review), which we complement here with agreeing autosomal DNA evidence.

Why did we (and others) not detect similarly strong signals of genetic admixture in U.S. Europeans, in contrast to U.S. Africans and U.S. Hispanics? One explanation may be that admixed individuals traditionally self-classify in a biased way and towards only one of the parental groups involved in the admixture process. Ancestry self-identification is the result of both visible traits (with a biological basis) such as skin color combined with cultural/sociological aspects (Bamshad and Guthery, 2007). In the present study rs1448484 appeared to be more informative and rs16891982 less informative for differentiating U.S. Africans from continental Europeans than continental Africans from continental Europeans. In contrast, rs16891982 was more informative for differentiating U.S. Europeans from continental Africans than continental Europeans from continental Africans. Rs1448484 is located within the *OCA2* gene, which when mutated can lead to oculocutaneous albinism type II (MIM# 203200); in addition, it has been previously associated with differences in pigmentation using pooled U.S. African / African-Caribbean population and U.S. European individuals (Shriver, et al., 2003). However, there is no evidence thus far that rs1448484 is directly involved in pigmentation variation, although it could be in LD with a functional OCA2 variant. In contrast, rs16891982 represents a non-synonymous amino acid change (F374L) in *SLC45A2,* and this gene, if mutated, leads to oculocutaneous albinism type IV (MIM# 606574). Notably, the *SLC45A2*-374 F allele of rs16891982 is almost fixed in Europeans (Soejima and Koda, 2007), and affects the amount of pigmentation (Stokowski, et al., 2007). Individuals carrying the genotypes *SLC45A2*-374L/L or *SLC45A2*-374L/F tend to show a darker skin color than *SLC45A2*-374F/F individuals (Cook, et al., 2009). Here we hypothesize that within the self-identified U.S. Europeans or U.S. Africans, individuals with the L/L or F/L genotypes would tend to declare themselves as U.S. African whereas F/F individuals would as U.S. Europeans. In that case, the presence of heterozygotes in U.S. Africans would decrease the *I_n_* statistic more than expected with continental Europeans and increase it between U.S. Europeans and continental Africans, as observed by our data. Although our data provide genetic evidence for the role of skin color in the complex process of ancestry self-identification, it would be extremely simplistic to reduce ancestry self- identification only to the type of analysis performed here.

## Figures and Tables

**Supp. Table S1:** Genotyping information autosomal SNPs

Multiplex A		PCR Primers	SBE Primers		Length	μM
rs1048610	F	AGGCAGGTCTCAGAACAATCC	GTGTGCTGCAGGGACCTTTC	F	20	5
	R	GTTCAGCATCGACATAGGGC				
rs1876482	F	GAGCTGTTGATAGAGCTTTTGTGG	ttttttGGCTGTACCCTCACTATTGGTG	R	28	5
	R	ACGTGACACATAAAGAAAATGCCAT				
rs2179967	F	AAGAGTGTGTTGTATGCTTTGGAAA	ttttttCTTTGGAAATGGGTGTGCAACA	F	28	6
	R	TCCTTCCAGCCCGACTAGAAC				
rs1858465	F	GATTTCAAAAAGTCTACAGATTTGG	tttttACTTCCTCTTTAATACTTCAACTGAGT	R	32	7
	R	TGACTTTGTCAAACTTCCTCTTTAA				
rs1371048	F	CTTAAATAGCCAAATAGCTCTAACT	ttttttttttATTTGAGTATGCTCTGTAGATGCTTC	R	36	5
	R	ACAAACGAAATATTTGAGTATGCT				
rs1369290	F	GAGGCCCTACATGACCTGTC	tttttttttttttttACCACAGGCTCTTGATAAAGTGTCT	F	40	5
	R	GGGCTCCTCTTTCGCTCA				
rs1465648	F	ACCAGAAGGAAAGAGAAAAAGCAC	tttttttttttttttttGAAAAAGCACAGTATCAAGTTTGACTT	F	44	6
	R	AACAAACTACAGCAACAGAATCTTT				
rs1391681	F	GAGTAGTTGCTCATGAAGCTGAAAA	ttttttttttttttttttttttTGTCACCCTTTACAAAACAGTTTGCA	F	48	5
	R	GGGCAGCCAAAAATAAAACAAAACA				
rs1461227	F	ACTGGGAAATTCTCACTGCAACT	tttttttttttttttttttttttttAACTACAACTAGCCCTAGGCTAATCTA	F	52	5
	R	TTGACAGATGGAGACACTGAAGC				
rs1907702	F	CCAACTCCTAATCAAGGCCTAC	ttttttttttttttttttttttttttttttCCTAATCAAGGCCTACAGAGACCTTC	F	56	5
	R	AGGAACATAAAGGAGGCCAGT				
rs2052760	F	ATTCAGAAAAGTGCATGCAGAAATT	ttttttttttttttttttttttttttttttttttATTATCAATGGGTTATTTTTGCCTCA	F	60	5
	R	GAGAGAGAGGAGTGAGAAAGGC				
rs1667751	F	CTGGTTCTTTTCCATCCAGCCTTTA	ttttttttttttttttttttttttttttttttttttttCTTTACAAGCTACAAGACTTACGCCT	F	64	5
	R	GAGATCACCAAGGGAGTAAGTACAG				

Multiplex B		PCR Primers	SBE Primers		Length	μM

rs1448484	F	TCTCCTTCCAAGCCTTCTGAAAAAT	tATGAGAGCTGGCAGCTTCC	F	20	6
	R	GCAACCACACAGAACACAGC				
rs714857	F	GAAACTTCCCTAATGGGTCTTGTGA	tttCTTGTGAACCTTGGCTCCCTG	F	24	6
	R	CCTCCCTCACACATAAAACTTCTCA				
rs16891982	F	ATCCAAGTTGTGCTAGACCAGAA	ttttttGAGGAAAACACGGAGTTGATGCA	F	29	5
	R	AGAGGAGTCGAGGTTGGATG				
rs1808089	F	TGTCAGGCCTTACCACTGCATAAGA	ttttttttACAAATGAGTAATGCCGTGGTGG	R	31	5
	R	AAACAACTCAGCGGCACAAA				
rs1478785	F	TCCTGGAGGCTTGAGGGCTA	tttttttttAGGGATGTTCATTTAAAATAACATCGC	F	36	5
	R	GGCTTGCTGGCTTTTTCTAGAT				
rs952718	F	GAGCCTAGATCCTGACTTCCTTG	tttttttttttttAAAATGCAAATTTCACCTTCTTCAAAT	R	40	5
	R	CTGTCACTGGAGATGTCATCTCAT				
rs1405467	F	AATTTGCAACAAAGAGGAAGGGGA	ttttttttttttttttttAAGTAGTCAGCTGAACTCACCTGAT	F	43	5
	R	GAGCAATAAGAGTGACTATGTCTGC				
rs1344870	F	CAATCTCAGTTTTAATTGCCATGT	ttttttttttttttttttttttTCGCTCTTAAGTATGTTTTCTTGGTC	F	48	5
	R	AGGATGTATTGGGGCCTTTC				
rs3843776	F	AGGCCACTGTTGTGGTTTATG	tttttttttttttttttttttttttttTGTTGTGGTTTATGTTTCACTTCGAC	F	53	6
	R	TGAGGGCTCTACAACACTGC				
rs721352	F	TCTGTGCCCAGATGCAAATCCTTA	tttttttttttttttttttttttttttttTGCTTGATGGCTCCACCTATCA	R	51	6
	R	GACCCAGAACTGTGCAGG				
rs722869	F	CCTTCTGCACTTGGGCATATT	tttttttttttttttttttttttttttttttttCAAATCCTTCATTTCACAAATGAAGCT	R	60	5
	R	AGGTAGAGATCTAACAAACCACAGT				
rs926774	F	AATCAAGTTCAGACTTTTGCCTCAT	tttttttttttttttttttttttttttttttttttttAAGCTATTGTAGTGAGGAAGGCTAGA	R	63	7

**Supp. Table S2:** MtDNA haplogroups observed among U.S. Americans and their assumed geographic region of origin

	Assumed continental origin			
mtDNA haplogroup	Asian	Eurasian	African	Native American
A	1			
A2				1
A5	1			
B2				1
B4a	1			
B4b1	1			
B4c	1			
B5b	1			
C1				1
D/E/G	1			
D/G	1			
D1				1
D4a	1			
D4e	1			
D4i	1			
D4k	1			
D5b	1			
E2	1			
F1a	1			
F1b	1			
F2a	1			
F3b	1			
G	1			
H		1		
H11		1		
H13a		1		
H1a		1		
H1b		1		
H1c		1		
H3a		1		
H5		1		
H6		1		
HV0		1		
I		1		
J1b		1		
J1c		1		
J2a		1		
K		1		
L0a			1	
L0a			1	
L1b			1	
L1c			1	
L2a1			1	
L2b			1	
L2c			1	
L2d			1	
L3			1	
L3a			1	
L3b			1	
L3d			1	
L3e1			1	
L3e2			1	
L3e3			1	
L3e4			1	
L3f			1	
L3h			1	
M10	1			
M35		1		
M7a	1			
M7b	1			
M8a	1			
M9a	1			
N1a		1		
N1b		1		
N9	1			
R*	0.5	0.5		
T1		1		
T2		1		
U2		1		
U3		1		
U4		1		
U5a		1		
U5b		1		
U6a			1	
U8a		1		
W		1		
X2		1		
X2a				1

**Supp. Table S3:** Genotyping information NRY SNPs

Additional	Haplogroup	SNP	Bibliogr aphical source	GenBank	dbSNPs accession (if known)	Position Y-chromosome	Forward Amplification primer (5′–> 3′)	Reverse Amplification primer (5′–> 3′)	concentration in PCR (μM)	Amplic on size (bp)	Minisequencing primers (target-specific sequence in capitals)	Orientation	concentration in miniseqreaction (μM)	Primer size (nt)	Mutation: Wildtype/Mutant**
hgE	E	M96	Refs	AC010889	rs9306841	20238386	GCCAGCCAAGAATGAAGAGA	TGAGCTGTGATGTGTAACTTGG	0.1	143	GGAAAACAGGTCTCTCATAATA	R	0.04	22	G/C
hgE	E1a	M33	2	AC009977		20199838	CCGTCATAGGCTGAGACAAGA	CCCCAAGAGAGACAACTGAC	0.15	150	ccacgtcgtgaaagtctgacaaCAGTTACAAAAGTATAATATGTCTGAGAT	R	0.06	51	C/G
hgE	E1b1	P2	3	AC010137		20070219	GAGAATCAGCTCCAGCCATC	TTTTGGATCTTCATGCTGGTT	0.03	100	gacaaAGGTGCCCCTAGGAGGAGAA	F	0.2	25	T/C
hgE	E1b1a	M2	6	AC011302	rs3893	12606580	ACGGAAGGAGTTCTAAAATTCAGG	AAAATACAGCTCCCCCTTTATCCT	0.1	147	cacgtcgtgaaagtctgacaaTTCATTGTTAACAAAAGTCC	R	0.06	41	G/A
hgE	E1b1a4	M154	2	AC010889		20352065	AGGCTACAAATTAGTGCGACA	GAGGCACAGATACTTAAACCATTG	0.06	77	acaaGTTACATGGCCTATAATATTCAGTACA	R	0.03	31	G/A
hgE	E1b1a7	M191	2	AC004474	rs2032590	13529007	AAAAATGGAGTGTTTATCAGAGCTT	CCCAGACACACCAAAATATCTC	0.3	122	gaaagtctgacaaAAAATATCTCATATTTTCAT	R	0.25	33	A/G
hgE	E1b1b	M215	2	AC006376	rs2032654	13977218	TCAAACTGTTGGTAAATTTTAGAGAAA	CAGAAGCATCAGCTGGAACA	0.25	97	gtcgtgaaagtctgacaaCAGCTGGAACAGTTAGAAAG	R	0.15	38	C/T
hgE	E1b1b1	M35	2	AC009977	rs1179188	20201091	AGGGCATGGTCCCTTTCTAT	TCCATGCAGACTTTCGGAGT	0.2	96	actgactaaactaggtgccacgtcgtgaaagtctgacaaTCGGAGTCTCTGCCTGTGTC	R	0.06	59	G/A
hgE	E1b1b1a	M78*	2	AC010889		20352691	TGCATTACTCCGTATGTTCGAC	TGGAAGCTTACCATCTTTTTATGA	0.05*	132	aagtctgacaaCTTATTTTGAAATATTTGGAAGGGC	R	0.02	36	A/C
hgE	E1b1b1a1	V12	7	AC012068		6883099	CTGAGTTGGATTGTTTTAAGTTGA	TTGGTCTCTCTTCATGTGCTG	0.15	150	acaaTTGTGTAGATAATTCAAAGT	R	0.25	24	C/T
hgE	E1b1b1a1a	M224	2	AC010889		20352687	TGCATTACTCCGTATGTTCGAC	TGGAAGCTTACCATCTTTTTATGA	0.05*	132	cgtgaaagtctgacaaAATTGATACACTTAACAAAGATACTTC	F	0.15	43	A/G
hgE	E1b1b1a1b	V32	7	AC012068		6992821	GCAAATGTTCCATGAATGGTG	CCAGCCAGAGAGGCACTTTA	0.4	111	CCCaactgactaaactaggtgccacgtcgtgaaagtctgacaaCACACATGTATATACACACC	R	0.25	63	C/G
hgE	E1b1b1a2	V13	7	AC012068		6902263	CAACAGTGGAGGACAAAGCA	AAGACCAGCCTGACCAACAT	0.15	106	cgtcgtgaaagtctgacaaGCTCAAACTTCCCTTG	R	0.15	35	A/G
hgE	E1b1b1a3	V22	7	AC012068		6919957	TGGCAATGCCTCAACTTACA	ATTCCCCAAGGTTTCAGAGG	0.15	110	CaactgactaaactaggtgccacgtcgtgaaagtctgacaaCCAAGGTTTCAGAGGTC	R	0.15	58	C/G
hgE	E1b1b1b	M81	2	AC010889	rs2032640	20351960	GCACTATCATACTCAGCTACACATCTC	TTGTTTCTTCTTGGTTTGTGTGA	0.03	99	acaaCTTGGTTTGTGTGAGTATACTCTATGAC	R	0.03	32	G/A
hgE	E1b1b1c	M123	2	AC010889		20223974	GTTGCCCAGGAATTTGCAT	CACAGAGCAAGTGACTCTCAAAG	0.15	89	taaactaggtgccacgtcgtgaaagtctgacaaCATTTCTAGGTATTCAGGCGATG	F	0.1	56	T/G
hgE	E1b1b1d	M281	4	AC010889	rs13447370	20223888	AGCAAAGTTGAGGTTGCACA	TGGGCAACACCAGAATCTAA	0.15	93	gtgccacgtcgtgaaagtctgacaaGCACAAACTCAGTATTATTAAAC	F	0.06	48	T/C
hgE	E1b1b1e	V6	3	AC012068		6992007	GATGGCACAGTGTTCGACAG	CTTCTCTCCAAATGCCTGCT	0.4	102	taggtgccacgtcgtgaaagtctgacaaCCTGCTGCCGCATCTGCA	R	0.02	46	T/C
hgE	E2	M75	2	AC010889	rs2032639	20349565	TGACTTGTCAAAAGCCAAAACA	TTGAACAGAGGCATTTGTGA	0.1	123	taggtgccacgtcgtgaaagtctgacaaGAAAAGACAATTATCAAACCACATCC	F	0.1	54	C/T

**Supp. Table S4:** NRY DNA haplogroups observed among U.S. origin Americans and their assumed geographic region of origin

	Assumed continental origin			
NRY haplogroup	Asian	Eurasian	African	Native American
A			1	
B			1	
C	1			
D	1			
E1a			1	
E1b1a*(xE1b1a4,E1b1a7)			1	
E1b1a7			1	
E1b1b1*(xE1b1b1a,E1b1b1b,E1b1b1c,E1b1b1d,E1b1b1e)		0.5	0.5	
E1b1b1a*(xE1b1b1a1,E1b1b1a2,E1b1b1a3)		0.5	0.5	
E1b1b1a1*(xE1b1b1a1a,E1b1b1a1b)		0.5	0.5	
E1b1b1a2		1		
E1b1b1a3		0.5	0.5	
E1b1b1b		1		
E1b1b1c		0.8	0.2	
E2			1	
G		1		
I		1		
J*(xJ2)		1		
J2		1		
K*(xL,M1,NO,P)	0.333	0.333	0.333	
N1c		1		
O	1			
Q1a				1
R1a		1		
R1b1b2		1		
R2		1		

## SUPP. METHODS

### Genotyping information autosomal SNPs

#### PCR and extension primer design

The 24 autosomal ASM SNPs were genotyped using two 12plex SNaPshot assays based on the principle of primer extension. 24 PCR primer pairs were selected using the commercially available primer selection software Visual OMP (DNA Software, Inc., Ann Arbor, MI). Template sequences consisting of approximately 500 base pairs up- and downstream from each SNP site were input into the Visual OMP program. Regions 30 bases up- and downstream from the SNP site were excluded from being selected as PCR primer binding sites. The size of each amplicon was kept under 150 base pairs to increase success when typing degraded samples e.g. in future forensic analyses. Each primer pair was selected independently (i.e. singleplex primer design). The final set of 24 PCR primer pairs were screened using AutoDimer for potential secondary structures such as primer-dimer and hairpin interactions (Vallone and Butler, 2004). Compatible primer pairs were divided into two separate PCR multiplexes containing 12 loci (see Table 1). The 24 extension primers were selected using the software module ‘ASPE tool’ (http://yellow.nist.gov:8444/dnaAnalysis/aspeToolsPage.do) present in the web-based AutoDimer software package (http://yellow.nist.gov:8444/dnaAnalysis/index.do). The user input consisted of the PCR amplicon sequences containing the corresponding SNP sites. Design parameter variables consisted of the desired length and predicted T_m_ of an extension primer. Primer sequences up- and downstream adjacent to the SNP site were selected that had the appropriate length and T_m_ characteristics. Extension primers were selected that had a predicted T_m_ of approximately 60 °C. Extension primers were subsequently screened for hairpin and primer-dimer interactions as described for the multiplex PCR primers. Poly-T tails of various lengths were added to the 5′ end of extension primers to allow sufficient fragment separation on a capillary electrophoresis system (see Table 1). All oligonucleotides were purchased from Qiagen Operon (Alameda, CA). Oligonucleotides were delivered lyophilized and desalted and stock solutions of 100 μM were prepared by adding in the appropriate volumes of a low salt buffer (10 mM TrisHCl and 0.1 mM EDTA pH 7.2)

#### Multiplex PCR

PCR conditions for each of the two 12plex amplification reactions were identical. Multiplex amplifications were carried out in a total volume of 15 μL. Approximately 1 ng of human template (genomic) DNA was present in the multiplex PCR amplifications. Final PCR reagent concentrations were: 1 unit of AmpliTaqGold® DNA polymerase (Applied Biosystems), 1x Taq Gold PCR buffer, 250 μM dNTPs (Promega Corp., Madison, WI), 2 mM Mg++, 0.16 mg/mL bovine serum albumin (BSA) fraction V (Sigma, St. Louis, MO), 0.4 μM of each amplification primer pair (24 primers per multiplex). Thermal cycling for PCR and SNaPshot assays was carried out using the GeneAmp 9700 (Applied Biosystems) running in 9600-emulation mode (i.e. ramp speeds of 1 °C/s). Note that for locus rs1344870 the final primer pair concentration was increased to 0.8 μM to reach balanced signals. The multiplex PCR thermal cycling conditions were as follows: 95°C for 10 min followed by 32 cycles of #95°C for 30 s, 55°C for 35 s, 72°C for 30 s# and a final step of 72°C for 7 min (afterwards incubated at 4°C). A combination of Exonuclease I (Exo I) and Shrimp Alkaline Phosphatase (SAP) (USB Corp., Cleveland, OH) was used to remove excess PCR primers and degrade unincorporated dNTPs. A mix of 1.4 μL of Exo I(1 μL = 10 units) and 2.6 μL SAP (1 μL = 1 unit) per sample was prepared and mixed. Four μL of the cocktail was added to each PCR reaction. The samples were incubated at 37°C for 90 min followed by 80°C for 20 min. The extensive incubation time ensured that the PCR primers were completely digested.

#### Multiplex primer extension reaction

Multiplex primer extension reactions were carried out in a total volume of 10 μL. Reaction components were: 2.5 μL of ABI Prism® SNaPshot® multiplex kit mix (Applied Biosystems), 0.5 μL of 10X AmpliTaqGold® PCR buffer, 3 μL of multiplex PCR products, 2.5 μL of deionized water, and 1.5 μL of a stock solution of extension primers (an unbalanced stock solution contained ∼5 μM of each extension primer, see Table 1 for the exact values). Thermal cycling conditions for extension reactions were carried out as described in the SNaPshot multiplex kit manual: 25 cycles of 96°C for 10 s, 50°C for 5 s, 60°C for 30s. Excess fluorescently labeled ddNTPs were inactivated by addition of 1 unit of Shrimp Alkaline Phosphatase (SAP). Reactions were mixed briefly and incubated at 37°C for 30 min then 80°C for 20 min. The ABI PRISM® 3130XL Genetic Analyzer was used for capillary electrophoresis (CE) with filter set E5 from the 5 dyes dR1 10, dR6G, dTAMRA™, dROX™, and LIZ™ after an appropriate spectral matrix had been created using materials from the matrix standard set DS-02 (Applied Biosystems). Fluorescently labeled extension reactions were prepared for CE analysis by mixing 14 μL of Hi-Di formamide™ (Applied Biosystems), 0.4 μL of the LIZ-120 internal sizing standard (Applied Biosystems), and 0.9 μL of SAP treated extension reaction. A 36 cm capillary array filled with denaturing POP6 performance optimized polymer (Applied Biosystems) was utilized for DNA fragment separation. A.C.E.™ (Ameresco, Solon, OH) capillary electrophoresis running buffer was used in 1 x concentration. Typical run module parameters were: Run temp = 60 °C, Capillary fill volume =184 steps, Pre run voltage =15 kV, Pre run time = 60 sec, Injection Voltage = 1kV, Injection time = 13 sec, Run Voltage = 15 kV, Data Delay = 200 sec, and Run time = 1200. Data analysis was performed using GeneMapperIDv3.2 software (Applied Biosystems). Bins and panels for the SNPs in each multiplex were developed based on fragment size and dye color for automated allele calling and are made available via the STRbase website http://www.cstl.nist.gov/biotech/strbase/SNP.htm.

Supp. Table S5. Genotype data for 24 ancestry-sensitive SNPs together with NRY and mtDNA haplogroup data for U.S. Americans

See extra excel file Supp. Table S5, available as additional online Supporting Information for this article.
